# A nonsense mutation in *PRNP* associated with clinical Alzheimer's disease^☆^

**DOI:** 10.1016/j.neurobiolaging.2014.05.013

**Published:** 2014-11

**Authors:** Rita Guerreiro, José Brás, Aleksandra Wojtas, Rosa Rademakers, John Hardy, Neill Graff-Radford

**Affiliations:** a Department of Molecular Neuroscience, UCL Institute of Neurology, University College London, London, England; b Department of Neuroscience, Mayo Clinic, Jacksonville, FL, USA; c Department of Neurology, Mayo Clinic, Jacksonville, FL, USA

**Keywords:** Alzheimer's disease, Prion, *PRNP*, Nonsense mutation, Exome sequencing

## Abstract

Here, we describe a nonsense haplotype in *PRNP* associated with clinical Alzheimer's disease. The patient presented an early-onset of cognitive decline with memory loss as the primary cognitive problem. Whole-exome sequencing revealed a nonsense mutation in *PRNP* (NM_000311, c.C478T; p.Q160*; rs80356711) associated with homozygosity for the V allele at position 129 of the protein, further highlighting how very similar genotypes in *PRNP* result in strikingly different phenotypes.

## 1 Introduction

Alzheimer's disease (AD) is a complex disorder with some cases known to be caused by mutations in 3 genes: the amyloid precursor protein (*APP*), Presenilin 1 (*PSEN1*), and Presenilin 2 (*PSEN2*). The Apolipoprotein E E4 allele increases the risk of AD by 3- to 15-fold, although several genetic loci (*CLU*, *PICALM*, *CR1*, *BIN1*, *MS4A*, *CD2AP*, *CD33*, *EPHA1*, *ABCA7*, *CD2AP*, *HLA-DRB5/DRB1*, *SORL1*, *PTK2B*, *SLC24A4*, *ZCWPW1*, *CELF1*, *FERMT2*, *CASS4*, *INPP5D*, *MEF2C*, *NME8*) have a low effect on disease risk (Guerreiro et al., 2013a). More recently, the application of exome sequencing to large cohorts of AD cases and healthy controls led to the identification of rare heterozygous variants in *TREM2* and *PLD3* as medium effect risk factors for the disease (Cruchaga et al., 2014, Guerreiro et al., 2013b, Jonsson et al., 2013).

The application of this technology to the study of small families and individual cases with different forms of dementia has also resulted in the association of unexpected molecular causes to different clinical phenotypes (for a review see, Guerreiro et al., 2014). For example, *TREM2* homozygous mutations, known to be the cause of Nasu-Hakola disease, were recently found to also cause frontotemporal dementia with no associated bone phenotypes (Guerreiro et al., 2013c); homozygous mutations in *ATP13A2* (a gene known to cause Kufor-Rakeb) and *GRN* (where heterozygous mutations cause frontotemporal dementia) were identified in families with neuronal ceroid-lipofuscinosis (Bras et al., 2012a, Smith et al., 2012). Exome sequencing has not only allowed the identification of the genetic causes of disease in cases that otherwise would have never been screened for mutations in the implicated genes because of their atypical phenotypes, but has also uncovered common biological pathways between different clinical entities (Bras et al., 2012b).

Here, we describe one more of these cases: a patient clinically diagnosed with AD found by exome sequencing to harbor a nonsense mutation in the *PRNP* gene.

## 2 Methods

When genetic tests for *APP*, *PSEN1*, and *PSEN2* revealed no mutations, the patient was included in a whole-exome sequencing study. Genomic DNA was prepared according to Illumina's TruSeq Sample Preparation v3 (Illumina, CA, USA) and capture was performed with Illumina's TruSeq Exome Enrichment according to the manufacturer's instructions. Sequencing was performed in Illumina's HiSeq2000 using 100 bp paired-end reads. Sequence alignment and variant calling were performed against the reference human genome (UCSC hg19) using bwa (Li and Durbin, 2009) and reads processed according with the Genome Analysis Toolkit best practices (McKenna et al., 2010). Variants were called using UnifiedGenotyper and recalibrated using VQSR, both tools from the GATK. Finally, variants were annotated using snpEff (Cingolani et al., 2012). The *PRNP* mutation was confirmed by Sanger sequencing using standard methodology.

## 3 Results

The analysis of the exome sequencing data confirmed the absence of pathogenic mutations in *APP*, *PSEN1*, and *PSEN2*. Additionally, no coding variants were found in the dementia associated genes *APP*, *PSEN2*, *GRN*, *TREM2*, or *PLD3*. The patient was found to carry the *PSEN1* (NM_000021) p.E318G and the *MAPT* (NM_001123066) p.Q230R variants.

Further inspection of the 9423 coding variants found (445 of which were novel), revealed a nonsense mutation in *PRNP* (NM_000311, c.C478T; p.Q160*; rs80356711) associated with homozygosity for the V allele at position 129 of the protein.

The patient was followed in the Mayo Clinic and presented an early-onset of cognitive decline at 38 years with memory loss as the primary cognitive problem, but also showing an impulsive behavior on her neuropsychological assessment. Her mother had a similar problem, also of early onset (no DNA was available for testing). Her maternal grandparents lived long and were said not to be affected. Her brother and daughter were also unaffected at the time of evaluation. She had temporary diarrhea, which was thought to be related to the introduction of Aricept, and her positron emission tomography scan showed left frontal hypometabolism. The patient was diagnosed with clinical AD and no neuropathologic assessment was possible.

## 4 Discussion

The mutation here described (p.Q160*) has been previously reported in 2 other cases (Table 1) diagnosed with an Alzheimer-like dementia. The first case did not have a detailed clinical description and no pathologic findings were reported (Finckh et al., 2000). The second case was deeply phenotyped, and neuropathologic evaluation showed abundant limbic and neocortical neuritic plaque-like structures and neurofibrillary tangles consistent with a neuropathologic diagnosis of AD. Immunohistochemical studies also demonstrated PrP immunopositive deposits (Jayadev et al., 2011).

**Table 1:** Main characteristics of cases reported in the literature with *PRNP* nonsense mutations

Mutation	M129V poly	Gender	Origin	AAO (y)	AAD (y)	Clinical features	Pathology features	Presence of diarrhea	Family history	Reference
Y145* (rs80356710)		F	Japanese	38	59	Alzheimer type clinical course	Many amyloid plaques (PrP) and diffuse neuropil threads of paired helical filaments			Kitamoto et al. (1993)
Y145* (rs80356710) probably the same case described by Kitamoto et al.	M/M		Japanese	38	59	Slowly progressive dementia	Severe diffuse atrophy of the cerebrum and dilation of the lateral ventricles; amyloid deposits in parenchymal and leptomeningeal blood vessels and in the perivascular neuropil; neurofibrillary lesions		“Family history is not contributory”	Ghetti et al. (1996)
Q160* (rs80356711)	Proband: M/M Brother: M/V (mutation on the M allele)	M	Austrian	Proband: 32 Brother: 48		Slowly progressive dementia	No postmortem	Not noted	Brother and father also with dementia onset at 48 y and reported to have died at 60 y	Finckh et al. (2000)
Q160* (rs80356711)	Proband: M/V Mother: M/M	F		Proband: 39 Mother: 59	47	The clinical and initial pathologic features in both patients were strongly suggestive of AD	Proband: abundant limbic and neocortical neuritic plaque-like structures and NFTs, consistent with a neuropathologic diagnosis of AD. Immunohistochemical studies: PrP immunopositive deposits. Mother: severe neurofibrillary tangles and neuritic plaque pathology in frontal cortex and hippocampus. Classic Lewy bodies and alpha-synuclein immunopositive inclusions and neurites.	Nothing noted in the proband but present in the mother	Mother with the same disease	Jayadev et al. (2011)
Y163*	Mutation in the V allele			Fourth decade with cognitive problems and seizures starting on the fifth and sixth decade	Average of 57 (range 40–70)	Chronic diarrhea with autonomic failure and a length-dependent axonal, predominantly sensory, peripheral polyneuropathy.	PrP-amyloid deposition was seen throughout the peripheral organs, including the bowel and peripheral nerves. Neuropathologic examination at end stage demonstrated PrP deposition in the form of frequent cortical amyloid plaques, cerebral amyloid angiopathy, and tauopathy. A unique pattern of abnormal PrP fragments was seen in brain tissue.	Yes	Dominant trait	Mead et al. (2013)
Y163* probably the same family reported by Mead S, et al.						Clinical diagnosis of hereditary sensory and autonomic neuropathy: chronic diarrhea, profound autonomic failure, and predominantly axonal sensory peripheral neuropathy in early adulthood.	Extensive central nervous system prion protein deposition including cerebral amyloid angiopathy and secondary tauopathy. Abnormal prion protein deposition was also seen in the duodenum.	Yes	9 patients from 1 family	http://dx.doi.org/10.1136/jnnp.2010.226340.31
Y163* probably the same case as reported in previous entrance and by Mead, S et al.							PrPSc deposition in blood vessels and parenchyma.			Revesz et al. (2009)
D178fs*25 (described by the authors as: “2 bp deletion in codon 178 that causes a premature stop codon and additional variable 25 amino acid at C-terminal from the mutation site”).		F	Japanese	Proband: 26Mother: 48	Proband: alive Mother: 49	Panautonomic failure, sensory neuropathy and cognitive impairment	No brain autopsy available	Yes (frequent diarrhea and vomiting)	Mother and maternal grandfather with the same disease	Matsuzono et al. (2013)
Y226*	M/V (mutation on the V allele)	F	Dutch	54		Dementia, visual, and acoustic hallucinations	PrP amyloid deposits, PrP-CAA, focal tau accumulations, mild focal spongiosis; diffuse and severe amyloid angiopathy involving small to medium-sized arteries and arterioles of the cerebral and cerebellar cortex and leptomeninges.		Mother diagnosed with ‘‘probable CJD’’ on the basis of comparable symptoms and signs.	Jansen et al. (2010)
Q227*	M/V (mutation on the V allele)	F	Dutch	39	45	Clinically diagnosed with FTD, extrapyramidal signs	PrP amyloid deposits, neurofibrillary tangles, no spongiosis; GSS disease phenotype with numerous multicentric amyloid plaques and neurofibrillary lesions in the cerebral gray matter and the absence of PrP-CAA.		One of the father's sisters died at the age of 42 y with comparable symptoms.	Jansen et al. (2010)

Key: AAD, age at death; AAO, age at onset; AD, Alzheimer's disease; F, female; FTD, frontotemporal dementia; M, male; Poly, polymorphism; NFTs, neurofibrillary tangles; y, years.

In the literature, 6 different mutations in *PRNP* leading to a premature truncation of the protein can be found (Table 1). None of these cases was initially diagnosed with a prion disease. In fact, the proband's mother in the report by Jayadev et al. (2011) was also neuropathologically diagnosed as AD before immunochemical studies were performed.

Recently, Mead et al. (2013) described an unusual phenotype associated with a novel nonsense mutation in *PRNP*. The affected members of this family carried the p.129V-163* *PRNP* truncation haplotype and developed autonomic failure with chronic diarrhea and peripheral polyneuropathy in adulthood.

The different truncating mutations in *PRNP* appear to have some common features namely: prolonged clinical courses, atypical for prion diseases, severe neurofibrillary tangle pathology, and high levels of cerebral amyloidosis. However, it is remarkable that the simple removal of an extra 3 amino acids on the same haplotype (V129 background), consistently results in a very different phenotype: truncated PRNP at amino acids 160 or 163 present with a clear hippocampal involvement or an autonomic defect, respectively.

## Disclosure statement

The authors declare no competing financial or personal interests that can influence the presented work.

## References

[bib1] Bras J., Guerreiro R., Hardy J. (2012). Use of next-generation sequencing and other whole-genome strategies to dissect neurological disease. Nat. Rev. Neurosci..

[bib2] Bras J., Verloes A., Schneider S.A., Mole S.E., Guerreiro R.J. (2012). Mutation of the parkinsonism gene ATP13A2 causes neuronal ceroid-lipofuscinosis. Hum. Mol. Genet..

[bib3] Cingolani P., Platts A., Wang le L., Coon M., Nguyen T., Wang L., Land S.J., Lu X., Ruden D.M. (2012). A program for annotating and predicting the effects of single nucleotide polymorphisms, SnpEff: SNPs in the genome of Drosophila melanogaster strain w1118; iso-2; iso-3. Fly (Austin).

[bib4] Cruchaga C., Karch C.M., Jin S.C., Benitez B.A., Cai Y., Guerreiro R., Harari O., Norton J., Budde J., Bertelsen S., Jeng A.T., Cooper B., Skorupa T., Carrell D., Levitch D., Hsu S., Choi J., Ryten M., Hardy J., Trabzuni D., Weale M.E., Ramasamy A., Smith C., Sassi C., Bras J., Gibbs J.R., Hernandez D.G., Lupton M.K., Powell J., Forabosco P., Ridge P.G., Corcoran C.D., Tschanz J.T., Norton M.C., Munger R.G., Schmutz C., Leary M., Demirci F.Y., Bamne M.N., Wang X., Lopez O.L., Ganguli M., Medway C., Turton J., Lord J., Braae A., Barber I., Brown K., Passmore P., Craig D., Johnston J., McGuinness B., Todd S., Heun R., Kolsch H., Kehoe P.G., Hooper N.M., Vardy E.R., Mann D.M., Pickering-Brown S., Kalsheker N., Lowe J., Morgan K., David Smith A., Wilcock G., Warden D., Holmes C., Pastor P., Lorenzo-Betancor O., Brkanac Z., Scott E., Topol E., Rogaeva E., Singleton A.B., Kamboh M.I., St George-Hyslop P., Cairns N., Morris J.C., Kauwe J.S., Goate A.M. (2014). Rare coding variants in the phospholipase D3 gene confer risk for Alzheimer's disease. Nature.

[bib5] Finckh U., Muller-Thomsen T., Mann U., Eggers C., Marksteiner J., Meins W., Binetti G., Alberici A., Sonderegger P., Hock C., Nitsch R.M., Gal A. (2000). High frequency of mutations in four different disease genes in early-onset dementia. Ann. N.Y Acad. Sci..

[bib6] Ghetti B., Piccardo P., Spillantini M.G., Ichimiya Y., Porro M., Perini F., Kitamoto T., Tateishi J., Seiler C., Frangione B., Bugiani O., Giaccone G., Prelli F., Goedert M., Dlouhy S.R., Tagliavini F. (1996). Vascular variant of prion protein cerebral amyloidosis with tau-positive neurofibrillary tangles: the phenotype of the stop codon 145 mutation in PRNP. Proc. Natl. Acad. Sci. U.S.A.

[bib7] Guerreiro R., Bras J., Hardy J. (2013). SnapShot: genetics of Alzheimer's disease. Cell.

[bib8] Guerreiro R., Bras J., Hardy J., Singleton A. (2014). Next generation sequencing techniques in neurological diseases: redefining clinical and molecular associations. Hum. Mol. Genet..

[bib9] Guerreiro R., Wojtas A., Bras J., Carrasquillo M., Rogaeva E., Majounie E., Cruchaga C., Sassi C., Kauwe J.S., Younkin S., Hazrati L., Collinge J., Pocock J., Lashley T., Williams J., Lambert J.C., Amouyel P., Goate A., Rademakers R., Morgan K., Powell J., St George-Hyslop P., Singleton A., Hardy J. (2013). TREM2 variants in Alzheimer's disease. N. Eng. J. Med..

[bib10] Guerreiro R.J., Lohmann E., Bras J.M., Gibbs J.R., Rohrer J.D., Gurunlian N., Dursun B., Bilgic B., Hanagasi H., Gurvit H., Emre M., Singleton A., Hardy J. (2013). Using exome sequencing to reveal mutations in TREM2 presenting as a frontotemporal dementia-like syndrome without bone involvement. JAMA Neurol..

[bib11] Jansen C., Parchi P., Capellari S., Vermeij A.J., Corrado P., Baas F., Strammiello R., van Gool W.A., van Swieten J.C., Rozemuller A.J. (2010). Prion protein amyloidosis with divergent phenotype associated with two novel nonsense mutations in PRNP. Acta Neuropathol..

[bib12] Jayadev S., Nochlin D., Poorkaj P., Steinbart E.J., Mastrianni J.A., Montine T.J., Ghetti B., Schellenberg G.D., Bird T.D., Leverenz J.B. (2011). Familial prion disease with Alzheimer disease-like tau pathology and clinical phenotype. Ann. Neurol..

[bib13] Jonsson T., Stefansson H., Steinberg S., Jonsdottir I., Jonsson P.V., Snaedal J., Bjornsson S., Huttenlocher J., Levey A.I., Lah J.J., Rujescu D., Hampel H., Giegling I., Andreassen O.A., Engedal K., Ulstein I., Djurovic S., Ibrahim-Verbaas C., Hofman A., Ikram M.A., van Duijn C.M., Thorsteinsdottir U., Kong A., Stefansson K. (2013). Variant of TREM2 associated with the risk of Alzheimer's disease. N. Eng. J. Med..

[bib14] Kitamoto T., Iizuka R., Tateishi J. (1993). An amber mutation of prion protein in Gerstmann-Straussler syndrome with mutant PrP plaques. Biochem. Biophys. Res. Commun..

[bib15] Li H., Durbin R. (2009). Fast and accurate short read alignment with Burrows-Wheeler transform. Bioinformatics.

[bib16] Matsuzono K., Ikeda Y., Liu W., Kurata T., Deguchi S., Deguchi K., Abe K. (2013). A novel familial prion disease causing pan-autonomic-sensory neuropathy and cognitive impairment. Eur. J. Neurol..

[bib17] McKenna A., Hanna M., Banks E., Sivachenko A., Cibulskis K., Kernytsky A., Garimella K., Altshuler D., Gabriel S., Daly M., DePristo M.A. (2010). The Genome Analysis Toolkit: a MapReduce framework for analyzing next-generation DNA sequencing data. Genome Res..

[bib18] Mead S., Gandhi S., Beck J., Caine D., Gallujipali D., Carswell C., Hyare H., Joiner S., Ayling H., Lashley T., Linehan J.M., Al-Doujaily H., Sharps B., Revesz T., Sandberg M.K., Reilly M.M., Koltzenburg M., Forbes A., Rudge P., Brandner S., Warren J.D., Wadsworth J.D., Wood N.W., Holton J.L., Collinge J. (2013). A novel prion disease associated with diarrhea and autonomic neuropathy. N. Engl. J. Med..

[bib19] Revesz T., Holton J.L., Lashley T., Plant G., Frangione B., Rostagno A., Ghiso J. (2009). Genetics and molecular pathogenesis of sporadic and hereditary cerebral amyloid angiopathies. Acta Neuropathol..

[bib20] Smith K.R., Damiano J., Franceschetti S., Carpenter S., Canafoglia L., Morbin M., Rossi G., Pareyson D., Mole S.E., Staropoli J.F., Sims K.B., Lewis J., Lin W.L., Dickson D.W., Dahl H.H., Bahlo M., Berkovic S.F. (2012). Strikingly different clinicopathological phenotypes determined by progranulin-mutation dosage. Am. J. Hum. Genet..

